# Astaxanthin from *Haematococcus pluvialis* and *Chromochloris zofingiensis*: Biosynthetic Pathways, Engineering Strategies, and Industrial Prospects

**DOI:** 10.3390/md23120485

**Published:** 2025-12-18

**Authors:** Shufang Yang, Xue Lu, Jia Wang, Ye Liu, Man Nie, Jin Liu, Han Sun

**Affiliations:** 1 Department of Food Science and Engineering, College of Chemistry and Environmental Engineering, Shenzhen University, Shenzhen 518060, China; 2 Discipline of Chinese and Western Integrative Medicine, Jiangxi University of Chinese Medicine, Nanchang 330004, China; 3 Institute of New Materials and Advanced Manufacturing, Beijing Academy of Science and Technology, Beijing 100089, China; 4 College of Food Science and Engineering, Ocean University of China, Qingdao 266003, China; 5 Center for Algae Innovation & Engineering Research, School of Resources and Environment, Nanchang University, Nanchang 330031, China

**Keywords:** astaxanthin, ketocarotenoid metabolism, metabolic engineering, microalgal biorefinery, techno-economic assessment

## Abstract

Astaxanthin, a high-value keto-carotenoid with potent antioxidant and health-promoting properties, has gained global attention as a sustainable nutraceutical and biotechnological product. The green microalgae *Haematococcus pluvialis* and *Chromochloris zofingiensis* represent two promising natural producers, yet they differ markedly in physiology, productivity, and industrial scalability. This review provides a focused comparative analysis of these two species, emphasizing their quantitative performance differences. *H. pluvialis* can accumulate astaxanthin up to ~3–5% of dry biomass but typically reaches biomass densities of only 5–10 g L^−1^, whereas *C. zofingiensis* achieves ultrahigh biomass concentrations of 100–220 g L^−1^ under heterotrophic fed-batch fermentation, although its astaxanthin content is much lower (~0.1–0.5% DW). While *H. pluvialis* remains the benchmark for natural astaxanthin due to its exceptionally high cellular content, its thick cell wall, slow growth, and strict phototrophic requirements impose major cost and operational barriers. In contrast, *C. zofingiensis* exhibits rapid and flexible growth under heterotrophic, mixotrophic, or phototrophic conditions and can achieve ultrahigh biomass in fermentation, though its ketocarotenoid flux and astaxanthin accumulation remain comparatively limited. Meanwhile, a rapidly growing patent landscape demonstrates global technological competition, with major portfolios emerging in China, the United States, and Europe, spanning chemical synthesis, microbial fermentation, algal metabolic engineering, and high-density cultivation methods. These patents reveal clear innovation trends—ranging from solvent-free green synthesis routes to engineered microalgae and yeast chassis for enhanced astaxanthin production—which increasingly shape industrial development strategies. By synthesizing recent advances in metabolic engineering, two-stage cultivation, and green extraction technologies, this review identifies key knowledge gaps and outlines a practical roadmap for developing next-generation astaxanthin biorefineries, with an emphasis on scalable production and future integration into broader biorefinery frameworks. The findings aim to guide future research and provide actionable insights for scaling sustainable, cost-effective production of natural astaxanthin.

## 1. Introduction

Astaxanthin is a red-orange xanthophyll carotenoid found widely in nature—from microalgae and yeast to marine crustaceans and fish—where it imparts vibrant pigmentation [1]. Beyond its ecological role, astaxanthin is valued as a high-end nutraceutical and food additive due to its notable antioxidant, anti-inflammatory, photoprotective, immunomodulatory, and metabolic-regulatory properties. Global demand for natural astaxanthin has increased rapidly in recent years, largely driven by evidence supporting its benefits for cardiovascular, ocular, metabolic, and neuroprotective health. The market was projected to exceed USD 1.5 billion by 2020 [2], with natural astaxanthin production dominated primarily by microalgae farms in the United States, China, and India, while major commercial producers—including Cyanotech (Kailua-Kona, Hawaii, USA), Algatech/Algalif (Kibbutz Ketura, Hevel Eilot region, Israel–Reykjanesbær, Iceland), and Yunnan Alphy Biotech (Chuxiong City, Yunnan Province, China)—supply a substantial share of the global market. This carotenoid aligns with the growing trend in functional foods and preventive health, being incorporated into supplements, cosmetics, and aquafeeds. However, over 95% of commercially available astaxanthin is still produced synthetically from petrochemical precursors via multi-step chemical synthesis—most commonly the Wittig reaction–based process—rather than through biological cultivation [2]. There is growing interest in sustainable, natural sources of astaxanthin—particularly because the (3S,3′S) isomer derived from algae is not only more bioactive but also, in many regions, the only form approved for human consumption. The price gap between synthetic and natural astaxanthin is likely to narrow as production technologies for natural astaxanthin continue to improve and scale up, while the regulatory gap is expected to persist due to stricter approval requirements and consumer preference for natural products. This has spurred research into alternative “astaxanthin biofactories” and improved production technologies. Astaxanthin’s chemical structure—featuring extended conjugated double bonds and polar β-ionone ring substituents—endows it with exceptional antioxidant capacity [3]. It is widely recognized as one of the most potent naturally occurring antioxidants, noted for its exceptional ability to neutralize reactive oxygen species and protect cellular components from oxidative damage. Quantitatively, its free-radical quenching activity has been measured to be an order of magnitude higher than that of other common carotenoids such as β-carotene, lutein, zeaxanthin or canthaxanthin, and on the order of a hundred-fold greater than α-tocopherol (vitamin E) [4].

Meeting the growing demand for natural astaxanthin hinges on efficient biological production. Among the diverse astaxanthin-producing microalgae reported to date, *Haematococcus pluvialis* and *Chromochloris zofingiensis* were selected as the primary comparative models in this review because they represent two fundamentally distinct yet industrially relevant production paradigms. *H. pluvialis* is the established benchmark organism, characterized by exceptionally high cellular astaxanthin content under photoinduced stress, and has underpinned most commercial natural astaxanthin production to date. In contrast, *C. zofingiensis* has emerged as a next-generation production platform that supports ultrahigh biomass accumulation under heterotrophic or mixotrophic conditions, albeit with substantially lower cellular astaxanthin content. Other astaxanthin-producing microalgae, such as Coelastrella spp., have also been reported; however, their cellular astaxanthin contents are generally lower (around 0.3% of dry weight) and biomass concentrations typically remain near ~1 g L^−1^, and robust industrial-scale production systems have not yet been established [5].

*H. pluvialis* is renowned for its extraordinary astaxanthin content under stress—it can accumulate astaxanthin up to ~5% of dry weight (DW) [6], with astaxanthin esters comprising 85–95% of total carotenoids in the cell [2]. The astaxanthin produced by *H. pluvialis* is predominantly the all-trans (3S,3′S) isomer and is mostly stored as mono- and diesters in cytosolic oil globules, which confers high stability and bioactivity. This alga’s natural astaxanthin is considered the industry “gold standard.” Nevertheless, *H. pluvialis* has a slow growth cycle and relatively low biomass productivity. In commercial two-stage cultivation (a green growth phase followed by a “red” stress phase), *H. pluvialis* culture densities are typically below 10 g L^−1^, and astaxanthin productivities on the order of 8–10 mg·L^−1^·day^−1^ are achieved over a week or more [2]. Such yields are modest, and the process is energy-intensive due to requirements for high light and specific stress conditions (e.g., nutrient starvation) to trigger astaxanthin synthesis. Moreover, *H. pluvialis* forms non-motile aplanospores with thick, sporopollenin-rich walls during astaxanthin accumulation, making cell disruption and pigment extraction difficult [7]. This necessitates costly processes and limits bioavailability unless cells are broken. Large-scale outdoor cultures are also prone to contamination and collapse; fungal infections, particularly by chytrid pathogens, can cause total culture loss. These issues—slow growth, sensitive culture conditions, recalcitrant biomass, and high contamination risk—keep *H. pluvialis*-derived astaxanthin expensive (US$3000–7000 per kg) [2] and constrain supply, motivating the search for more robust production systems.

*Chromochloris zofingiensis*, an oleaginous green microalga, has emerged as the most promising “next-generation” astaxanthin producer to complement or replace *H. pluvialis*. Formerly known as a strain of *Chlorella*, *C. zofingiensis* is capable of both photosynthetic (autotrophic) growth and heterotrophic growth on organic carbon, giving it great flexibility in cultivation [8]. Under nutrient-replete conditions it accumulates lipids (up to 50% DW triacylglycerol) rather than carotenoids, but it can redirect metabolism to astaxanthin under stress cues similar to *H. pluvialis* (high light, oxidative stress, etc.) [9]. A key advantage of *C. zofingiensis* is its rapid growth to very high cell densities in fermentation. Using glucose-fed heterotrophic cultivation, *C. zofingiensis* can reach cell concentrations on the order of 100 g L^−1^ within days–orders of magnitude higher biomass than typical *H. pluvialis* cultures. For example, Zhang et al. achieved ~98 g L^−1^ in a 14-day fed-batch run [10], and recent process optimizations have pushed the biomass to an impressive 180–220 g L^−1^ in under 10–12 days in 7.5 L and 500 L fermenters [11]. Such high-density fermentation is not feasible for *H. pluvialis*, which lacks glucose utilization and experiences severe light self-shading at high cell densities. In contrast, *C. zofingiensis* grows robustly in dark, oxygenated fermentors, even on low-cost substrates such as cane molasses or other food-industry byproducts. The trade-off is that heterotrophic *C. zofingiensis* cells initially accumulate relatively little astaxanthin (often <0.1% DW) [11].

This review provides a systematic and integrated comparison of *H. pluvialis* and *C. zofingiensis* across the entire pipeline of natural astaxanthin production, highlighting the core trade-off between the exceptionally high astaxanthin content of *H. pluvialis* and the ultrahigh biomass output achievable with *C. zofingiensis*. We examine differences in their metabolic pathways for astaxanthin biosynthesis, including carotenoid precursor formation and the roles of β-carotene ketolase and hydroxylase enzymes in each alga, as well as how astaxanthin is sequestered (e.g., esterification into lipid droplets). Key engineering aspects are compared: *H. pluvialis*’s strictly photoautotrophic, stress-dependent cultivation versus *C. zofingiensis*’s two-stage heterotrophic-plus-phototrophic process, and how these affect productivity, energy usage, and contamination risk. Recent technological innovations are also surveyed—for example, genetic engineering approaches like CRISPR/Cas9 to modify lipid metabolism genes and enhance astaxanthin storage, optimized fed-batch nutrient feeding and C/N ratio control to boost yields, and the adoption of “green” extraction methods like supercritical CO_2_ (SFE-CO_2_) for solvent-free pigment recovery. In addition to biological and engineering perspectives, this review uniquely integrates recent techno-economic analyses and life-cycle assessments to evaluate the cost, sustainability, and industrial viability of both species. By synthesizing these multidisciplinary advances into a unified comparative framework, the review identifies critical knowledge gaps, highlights species-specific bottlenecks and opportunities, and proposes a forward-looking roadmap toward next-generation astaxanthin biorefineries capable of supporting large-scale, sustainable production.

## 2. Astaxanthin: Structure, Functions, and Market Demand

### 2.1. Chemical Structure and Stereoisomers

Astaxanthin is a keto-carotenoid classified among tetraterpenes, with the molecular formula C_40_H_52_O_4_ and a molecular weight of approximately 596.85 Da. Its structure consists of a polyene backbone containing 13 conjugated double bonds flanked by two terminal β-ionone rings, each bearing both a hydroxyl (–OH) and a keto (C=O) group. This configuration results in a polar–nonpolar–polar amphiphilic structure, enabling astaxanthin to span lipid bilayers and anchor at both polar ends, thus providing superior membrane protection compared to other carotenoids [3].

**Figure 1:**
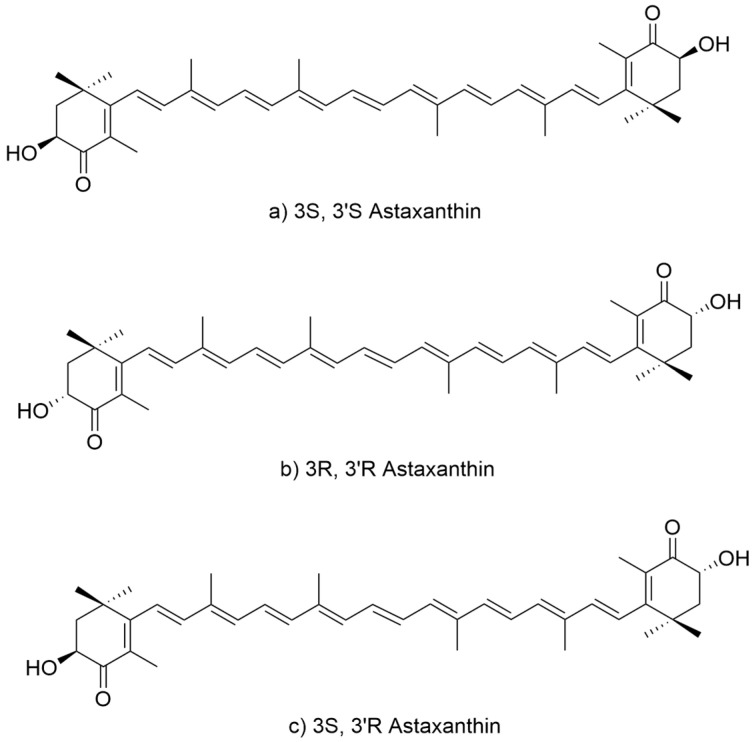
Structure of astaxanthin stereoisomers. (**a**) 3S,3′S form, (**b**) 3R,3′R form and (**c**) 3R,3′S form.

Astaxanthin contains two chiral centers at C-3 and C-3′, giving rise to three stereoisomers: the enantiomers (3S,3′S) (Figure 1a) and (3R,3′R) (Figure 1b), and the meso form (3R,3′S) (Figure 1c), which is optically inactive. In nature, the two enantiomeric forms predominate depending on the biological source. Additionally, due to the seven conjugated double bonds, multiple cis/trans geometric isomers theoretically exist; however, the all-trans configuration is thermodynamically most stable and most frequently observed in natural samples. Naturally derived astaxanthin from microalgae such as *H. pluvialis* is predominantly found in the (3S,3′S), all-trans esterified form [12], which is attributed to the stereoselectivity of the endogenous carotenoid biosynthetic enzymes. The β-carotene hydroxylase and ketolase involved in astaxanthin formation exhibit strong enantioselectivity toward introducing S-configured groups at C-3 and C-3′, thereby favoring the biosynthesis of the (3S,3′S) isomer. This stereoisomer is also considered more conformationally stable and interacts more effectively with biological membranes, which may enhance its photoprotective and antioxidant functions in algal cells.

**Figure 2:**
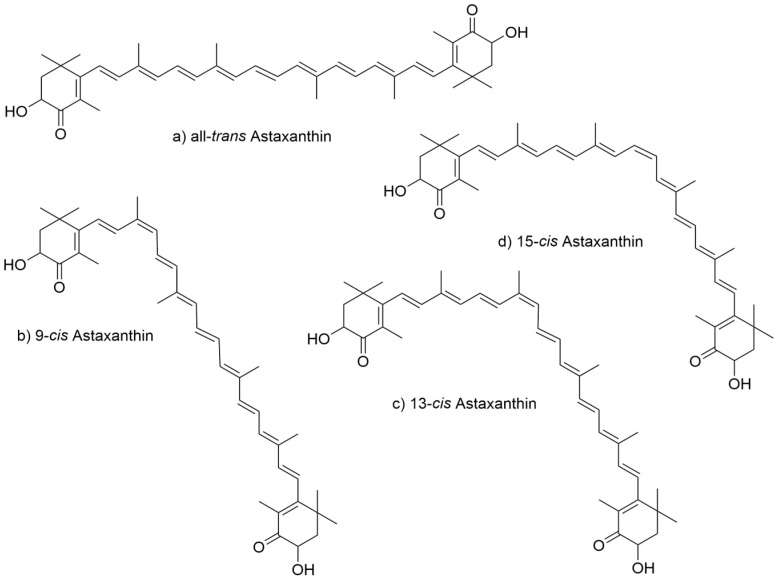
Structure of astaxanthin geometric isomers. (**a**) all-trans, (**b**) 9-cis, (**c**) 13-cis, (**d**) 15-cis astaxanthin.

In the long polyene chain of the astaxanthin molecule, each double bond can adopt either a cis (Z) or trans (E) configuration. In nature, astaxanthin typically exhibits cis configurations at positions 9, 13, and 15. The geometric isomers most commonly observed in natural astaxanthin include all-trans (Figure 2a), 9-cis, 13-cis, 15-cis, 9,13-cis, 9,15-cis, 13,15-cis, and 9,13,15-cis. Among these, the 9-cis (Figure 2b), 13-cis (Figure 2c), and 15-cis (Figure 2d) forms are the most frequently encountered, owing to steric hindrance (Figure 2). Because the branched methyl groups do not impose significant steric interference, the all-trans form of astaxanthin is regarded as the most stable, exhibiting the highest antioxidant activity and bioavailability.

### 2.2. Bioactivity and Health Applications

Astaxanthin’s unique molecular configuration confers exceptional antioxidant activity. It efficiently neutralizes singlet oxygen and other reactive oxygen and nitrogen species (ROS/RNS), thereby attenuating oxidative stress. Beyond antioxidation, astaxanthin exerts potent anti-inflammatory and immunomodulatory effects [13]. It inhibits inflammatory cytokine expression and enhances both humoral and cellular immunity, including the activation of T and B lymphocytes [14]. Astaxanthin is also neuroprotective, capable of crossing the blood–brain barrier and protecting neurons against oxidative damage and mitochondrial dysfunction. It has been shown to improve cognitive function and may delay neurodegenerative processes [13]. In the visual system, astaxanthin protects retinal cells by enhancing microcirculation and reducing photo-oxidative damage, with potential applications in age-related macular degeneration [15]. In metabolic and exercise physiology, astaxanthin mitigates oxidative stress during intense physical activity, reduces lactate accumulation, and accelerates recovery. It also promotes lipid utilization and inhibits adipogenesis, suggesting a role in metabolic regulation and weight management [13].

Recent clinical trials highlight astaxanthin’s benefits in cardiovascular, metabolic, and oxidative health, as well as cognitive function. In prediabetic adults with dyslipidemia, 12 mg d^−1^ for 24 weeks (*n* = 34) significantly lowered LDL (~0.33 mM) and total cholesterol (~0.30 mM) [16]. In heart failure patients (*n* = 80), high-dose astaxanthin (20 mg d^−1^, 8 weeks) raised antioxidant indices (SOD +157 U mL^−1^) and reduced lipid peroxidation markers (MDA –2.19 nmol L^−1^) vs placebo [17]. These trials also noted reductions in other risk markers (e.g., fibrinogen, uric acid). Cognitive outcomes are mixed: older adults (6–12 mg d^−1^, 12 weeks) showed improved memory test scores without statistical significance [18]. Collectively, its broad biological efficacy and established safety support the use of astaxanthin as a high-value functional nutraceutical in supplements, cosmetics, and animal nutrition.

### 2.3. Market Landscape and Production Technologies

The global market for astaxanthin has expanded rapidly in response to growing demand for natural antioxidants. Synthetic astaxanthin, derived from petrochemical precursors, still dominates the market due to its low cost and scalable production [12]. However, synthetic variants consist of racemic mixtures with inferior bioactivity and are not permitted in food or pharmaceutical applications in many countries.

Natural astaxanthin is primarily obtained through microbial fermentation or extraction from marine byproducts. Crustacean shells (e.g., shrimp, crab) contain minute quantities of astaxanthin, but extraction is technically challenging and commercially impractical. Microalgal production, particularly using *H. pluvialis*, is currently the most viable approach for high-purity, bioactive astaxanthin [19]. This species accumulates astaxanthin predominantly in the (3S,3′S) esterified form. Yeast-based production using *Phaffia rhodozyma* provides a cost-effective alternative [20]. However, it produces the (3R,3′R) form with a high proportion of *cis* isomers and lower antioxidant activity, limiting its applications primarily to animal feed.

The global astaxanthin market was valued at USD 1.94 billion in 2022 and is projected to reach USD 2.57 billion by 2025, with a compound annual growth rate (CAGR) exceeding 15% precedence research. Forecasts suggest continued growth, potentially surpassing USD 6.8 billion by 2034. Natural astaxanthin commands a premium price—∼$2500–7000/kg for *H. pluvialis*-derived pigment—compared to ∼$1000–2000/kg for synthetic grades [21]. Price variations reflect differences in purity, stability, isomeric composition, and regulatory approval. As process innovations improve production efficiency and consumer awareness increases, the market share for natural astaxanthin is expected to rise significantly in the coming decade.

At the global level, commercial astaxanthin production and utilization are concentrated in a limited number of countries. Industrial-scale *H. pluvialis* facilities have been established in China, Chile, India, Israel, Iceland, Japan, Sweden, and the United States, supporting the supply of natural astaxanthin for aquaculture, nutraceutical, and cosmetic applications [22]. From a market perspective, North America, Europe, and the Asia-Pacific region represent the major demand centers, with the United States, China, Japan, and several European countries being key consumers in high-value segments such as dietary supplements, functional foods, and skincare products [23,24]. Recent market analyses indicate that Asia-Pacific, particularly China, India, Japan, and South Korea, is expected to exhibit the most rapid growth, driven by increasing health awareness, expansion of aquaculture, and strong demand for premium cosmetic products, whereas North America and Europe are projected to maintain steady growth in natural astaxanthin utilization.

## 3. Biosynthetic Pathways of Astaxanthin

### 3.1. Haematococcus pluvialis

**Figure 3:**
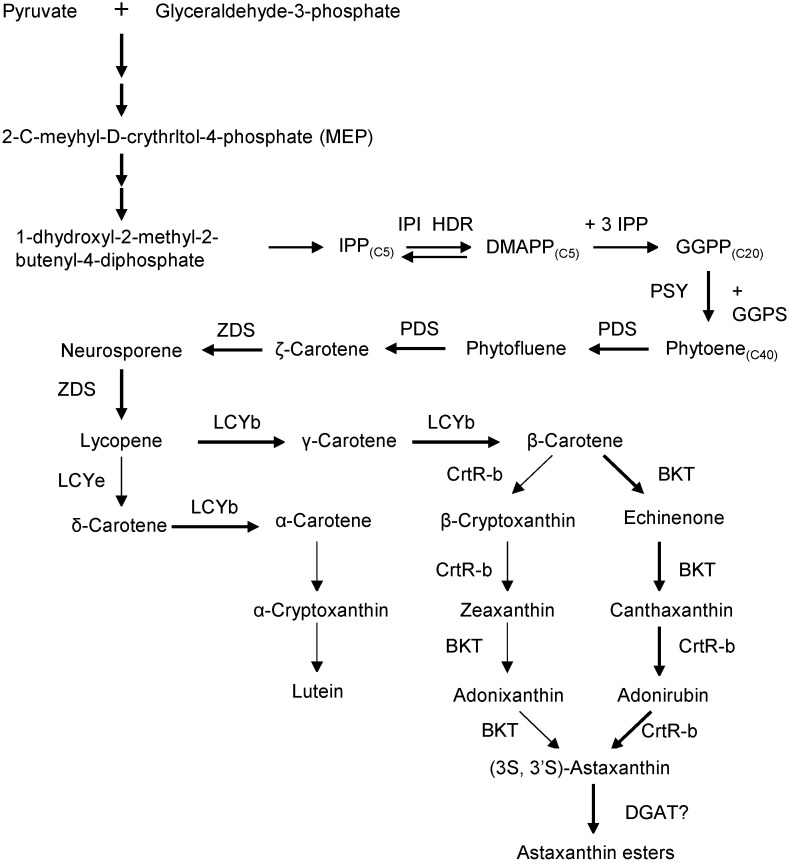
Biosynthesis of astaxanthin in *H. pluvialis*.

Astaxanthin biosynthesis in *H. pluvialis* occurs in three stages: (1) synthesis of β-carotene, (2) conversion of β-carotene to free astaxanthin, and (3) esterification of astaxanthin [25]. The process begins in the chloroplast via the MEP (2-C-methyl-D-erythritol 4-phosphate) pathway, which converts photosynthesis-derived pyruvate and glyceraldehyde-3-phosphate into isopentenyl pyrophosphate (IPP) (Figure 3). IPP is isomerized to dimethylallyl pyrophosphate (DMAPP), and one DMAPP plus three IPP molecules are then condensed by geranylgeranyl pyrophosphate synthase to form geranylgeranyl pyrophosphate (GGPP). The first committed step is catalyzed by phytoene synthase (PSY), which joins two GGPP molecules head-to-tail to produce the C_40_ carotenoid phytoene. Subsequent desaturation by phytoene desaturases (PDS) and ζ-carotene desaturase (ZDS) (with plastid terminal oxidases as cofactors) converts phytoene through colorless intermediates into the pink pigment lycopene. Lycopene cyclization follows: lycopene β-cyclase (LCYb) adds β-ionone rings, yielding β-carotene [25]. Under normal conditions, most flux goes toward β-carotene rather than α-carotene. The final steps are successive oxygenations of β-carotene. β-carotene ketolase (BKT) adds keto groups and β-carotene hydroxylase (CrtR-b) adds hydroxyls. In practice, *H. pluvialis* first ketolates β-carotene to canthaxanthin and then hydroxylates canthaxanthin to astaxanthin. BKT has higher affinity for β-carotene, so a keto group is usually introduced before the enantioselective hydroxylation of the resulting ketocarotenoids.

Following synthesis, free astaxanthin is transported to the endoplasmic reticulum (ER) for esterification. Under stress, *H. pluvialis* converts most astaxanthin into fatty-acid esters within cytosolic lipid bodies. The hydroxyl groups on the terminal rings of astaxanthin become mono- or di-esters with fatty acids (mainly palmitic C16:0, oleic C18:1, or linoleic C18:2) [26]. In mature red cyst cells, roughly 70% of astaxanthin is monoesterified and 25% is diesterified [27]. These modifications embed the polar ketocarotenoid in the nonpolar lipid droplet matrix. Although the exact acyltransferases remain unknown, diacylglycerol acyltransferases (DGATs) are strong candidates. In vitro assays show that ER fractions can esterify astaxanthin and that adding DGAT inhibitors greatly reduces ester formation [28]. Molecular docking predicts that *H. pluvialis* DGAT1 (HpDGAT1) has an astaxanthin-binding pocket, and overexpression of HpDGAT1 (in heterologous hosts) increases both total lipids and astaxanthin esters. These findings suggest HpDGAT1 may possess dual function in TAG synthesis and xanthophyll esterification [29].

### 3.2. Chromochloris zofingiensis

**Figure 4:**
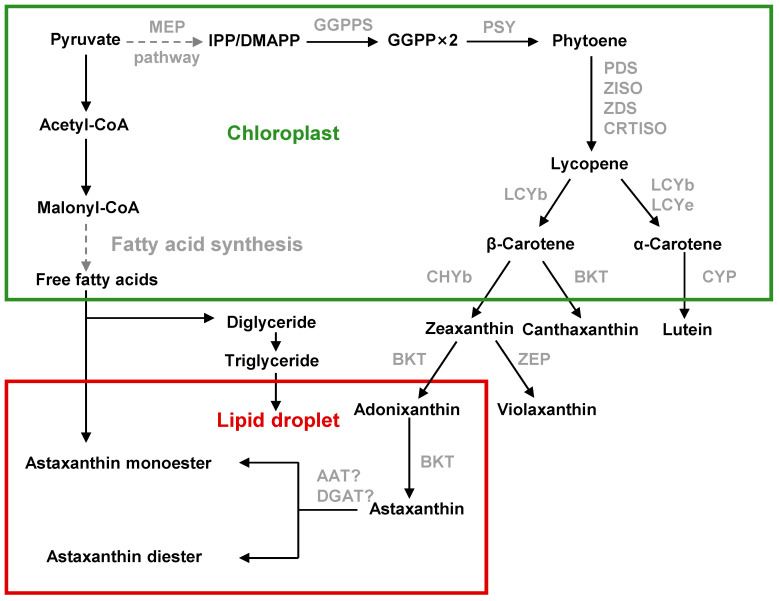
Astaxanthin biosynthesis pathway in *C. zofingiensis*.

*C. zofingiensis* follows a broadly similar pathway but with notable distinctions. Like *H. pluvialis*, *C. zofingiensis* uses the chloroplast-localized MEP pathway to produce IPP/DMAPP (Figure 4) [30]. Inhibitor studies confirm that carotenoid precursors derive from MEP rather than the cytosolic mevalonate route [31]. One DMAPP and three IPP units form GGPP, and two GGPP condense into phytoene, as in other algae. Phytoene is then desaturated (PDS, ZISO, ZDS, CRTISO) into lycopene [32]. The key rate-limiting enzyme PSY controls flux into carotenoids [31]. Lycopene cyclization by LCYb and LCYe determines the β- vs. α-carotene branch. Stress conditions strongly upregulate LCYb and suppress LCYe in *C. zofingiensis* [33], shifting flux toward β-carotene (the precursor of ketocarotenoids) at the expense of α-carotene.

Carotenogenesis in *C. zofingiensis* then diverges: β-carotene is hydroxylated by a non-heme di-iron β-carotene hydroxylase (CHYb) to form zeaxanthin, and ketolated by β-carotene ketolase (BKT) to form ketocarotenoids. Importantly, *C. zofingiensis* BKT efficiently ketolates zeaxanthin to astaxanthin via intermediates (adonixanthin), whereas CHYb cannot hydroxylate canthaxanthin to astaxanthin [30]. Thus, the pathway proceeds from β-carotene to zeaxanthin (catalyzed by CHYb), then to adonixanthin and finally to astaxanthin (catalyzed by BKT), which differs from that in *H. pluvialis*. As a result, *C. zofingiensis* accumulates canthaxanthin (approx. 30% of its secondary carotenoids) as a by-product.

Synthesis of these carotenoids is compartmentalized: primary carotenoids (phytoene through β-carotene, zeaxanthin) are made in the chloroplast, while the final ketolation steps occur outside the chloroplast. BKT is localized to the ER and CHYb to the chloroplast in *C. zofingiensis* [31]. Under astaxanthin-inducing stress, β-carotene and zeaxanthin are exported from the chloroplast. In fact, algal lipid droplets (LDs) physically connect chloroplasts and ER and serve as conduits: β-carotene and zeaxanthin move along the LD monolayer to the ER for BKT-mediated ketolation, and the ketolated carotenoids (astaxanthin, canthaxanthin, etc.) then diffuse back into LDs for storage [34]. As in *H. pluvialis*, stress-induced *C. zofingiensis* cells store most astaxanthin in esterified form. Indeed, up to ≈90% of total astaxanthin in *C. zofingiensis* is found as mono- or di-esters within cytosolic lipid droplets.

*C. zofingiensis* is an oleaginous alga: it can accumulate over 50% of DW as lipids (mostly triacylglycerol). The major fatty acids produced are palmitic acid (C16:0), oleic acid (C18:1), linoleic (C18:2) and linolenic (C18:3) acids [35]. These C16–C18 acyl chains serve as substrates for astaxanthin esterification. Notably, lipid and astaxanthin biosynthesis share the precursor pyruvate and are co-regulated. In *C. zofingiensis* both TAG and astaxanthin accumulate under the same stress conditions, and neither pathway strongly diverts carbon from the other under normal conditions.

### 3.3. Comparative Analysis

Although *C. zofingiensis* possesses the ability to synthesize astaxanthin, its synthesis level is far lower than that of *H. pluvialis*, likely due to differences in their biosynthetic pathways and regulatory mechanisms. Key differences in astaxanthin biosynthesis between *H. pluvialis* and *C. zofingiensis* are summarized in Table 1, including pathway regulation, enzymatic specificity, flux control, and storage mechanisms. Under stress conditions such as nitrogen limitation and high light, *H. pluvialis* exhibits upregulation of the MEP pathway and enhanced formation of lycopene from IPP/DMAPP, leading to an overall increase in carbon flux toward carotenoid biosynthesis; in contrast, *C. zofingiensis* shows no transcriptional upregulation of the MEP pathway, resulting only in a redistribution between primary and secondary carotenoids without increasing the total carbon flux, which restricts astaxanthin accumulation [36]. Furthermore, CHYb in *C. zofingiensis* lacks hydroxylase activity toward canthaxanthin, so astaxanthin synthesis relies solely on zeaxanthin ketolation, whereas *H. pluvialis* CHYb can catalyze canthaxanthin hydroxylation, allowing the organism to produce astaxanthin efficiently through both canthaxanthin hydroxylation and zeaxanthin ketolation without excessive intermediate buildup [31,37]. In addition, BKT in *C. zofingiensis* acts not only on β-carotene and zeaxanthin but also converts lutein into keto-lutein for accumulation, thereby diverting carotenoid flux away from astaxanthin synthesis [38]. Moreover, violaxanthin biosynthesis competes with astaxanthin for the substrate zeaxanthin, further reducing zeaxanthin availability for astaxanthin formation [39]. Finally, because *C. zofingiensis* accumulates other pigments such as keto-lutein and adonixanthin, its astaxanthin acyltransferase must process multiple esterification substrates, creating substrate competition that lowers astaxanthin esterification efficiency and subsequently feeds back to inhibit astaxanthin biosynthesis [38,40].

Growth characteristics differ. *H. pluvialis* grows slowly, has thick resistant cell walls, and is an obligate photoautotroph–it requires light induction to produce astaxanthin. By contrast, *C. zofingiensis* grows relatively fast and flexibly: it tolerates mixotrophic or heterotrophic cultivation on sugars, in addition to photoautotrophy. This makes *C. zofingiensis* easier to culture at high density, but its inherent metabolic limits cap its astaxanthin yield [41]. In engineering terms, efforts for *H. pluvialis* focus on improving light utilization, growth rate, and stress resilience (to boost biomass and pigment accumulation), whereas for *C. zofingiensis* targets include enhancing MEP-pathway flux (e.g., overexpressing DXS/DXR/HDR) and optimizing the CHYb/BKT enzymes to increase the ketocarotenoid flux [31].

## 4. Factors Affecting Astaxanthin Accumulation

### 4.1. Light

Light is a primary driver of astaxanthin biosynthesis in both *H. pluvialis* and *C. zofingiensis*. High irradiance triggers oxidative stress in the chloroplast, leading to enhanced carotenoid production as a photoprotective response. *H. pluvialis* is especially responsive: intense light (>200–300 μmol photons m^−2^ s^−1^) causes a rapid transition from green vegetative cells to orange-red cysts packed with astaxanthin [32]. For instance, raising the irradiance from ~15 to 160 μmol photons m^−2^ s^−1^ has been shown to increase the final astaxanthin concentration in *H. pluvialis* culture from only ~0.7 mg L^−1^ to ~6.5 mg L^−1^ [42], reflecting a strong intensity-dependent boost in carotenoid accumulation. In addition, suitable light wavelengths are particularly effective at inducing astaxanthin in *H. pluvialis* [43,44]. Experiments with mixed light spectra showed that blue light elevates ROS levels (e.g., increased malondialdehyde and catalase activity) and upregulates astaxanthin synthesis, whereas red light of similar intensity chiefly promotes photosynthetic growth [45]. Under a blue-to-white light ratio of 3:1, the system achieved a yield of 91.8 mg L^−1^, which was 15.8% higher than the white-light treatment and 11.8% higher than the blue-light condition [6]. This differential response is mediated by photoreceptors: a blue-light absorbing cryptochrome (HpCRY4) in *H. pluvialis* is upregulated under blue light, and its functional characterization confirmed that cryptochrome signaling promotes carotenoid biosynthetic genes and astaxanthin accumulation [46].

*C. zofingiensis*, similarly, accumulates astaxanthin under high light stress as a protective mechanism. In phototrophic culture it requires a sufficient light threshold to trigger secondary carotenoid formation, although its cells do not form thick-walled cysts as in *H. pluvialis*. However, excessively high irradiance can become counterproductive: astaxanthin biosynthesis was found to be substantially inhibited when light intensity was raised from ~150 to 300 μmol photons m^−2^ s^−1^ in *C. zofingiensis* [47]. A key distinction is the magnitude of response: *H. pluvialis* can devote an exceptionally large fraction of its carbon flux to astaxanthin, whereas *C. zofingiensis* reaches lower cellular astaxanthin content. For example, laboratory studies have shown that *C. zofingiensis* can accumulate approximately 4–6 mg g^−1^ of astaxanthin under combined high-light and stress conditions [31], and under optimized high-light plus nutrient-deprivation conditions it can reach ~7 mg g^−1^ [47], in contrast to *H. pluvialis* which can exceed 25 mg g^−1^ under similar conditions, or even ~46 mg g^−1^ with additional stimulants [22].

### 4.2. Temperature

Temperature profoundly affects growth and secondary metabolism in these microalgae. Both species thrive in a mesophilic range, with optimal growth around 20–28 °C. *H. pluvialis* shows maximal vegetative growth near ~23 °C, and this temperature also favors basal carotenoid levels [48]. When temperatures rise into the 30–33 °C range, *H. pluvialis* experiences heat stress that rapidly induces the astaxanthin-accumulating red cyst stage. Notably, exposure to ≥30 °C can trigger encystment and a 2–3-fold higher astaxanthin content (relative to 20 °C controls) within ~48 h [32]. This heat-induced carotenogenesis is attributed to thermal damage to the photosystems and enhanced generation of ROS at elevated temperature. However, acute heat stress also arrests cell division and can be lethal if too extreme or sudden, so gradual shifts are preferred to allow acclimation. Therefore, *H. pluvialis* growth follows a bell-shaped temperature response, with biomass sharply decreasing beyond 28 °C. Astaxanthin accumulation increases under moderate heat, peaking at 30–33 °C. *C. zofingiensis* has received less study regarding temperature optima and limits, but available data suggest a similar profile: its photosynthetic activity peaks at roughly 25–27 °C [49]. Severe heat stress in *C. zofingiensis* (≥35 °C) is likely deleterious, though *C. zofingiensis* might endure slightly higher temperatures than *H. pluvialis* without immediate cell death, owing to its generally robust growth traits (this remains to be verified experimentally). One report indicates *C. zofingiensis* astaxanthin content may peak around 24 °C, with declines at higher temperatures [49]. Therefore, *C. zofingiensis* growth increases with temperature up to approximately 24 °C, but declines as the temperature continues to rise.

Unlike *H. pluvialis*, *C. zofingiensis* does not undergo a dramatic cyst transformation, but heat stress still impairs its chlorophyll and promotes carotenoid accumulation to some extent. In both algae, moderate heat shock synergizes with light and nutrient stress to amplify astaxanthin synthesis. For instance, combining high light with elevated temperature has been shown to intensify carotenoid accumulation in other astaxanthin-producing algae by exacerbating ROS pressure [48]. Thus, temperature is a tunable parameter: maintaining the culture at an optimal temperature (~25 °C) supports rapid growth during the “green” stage, while a shift to supraoptimal but sub-lethal heat can serve as an additional stressor in the astaxanthin induction stage. Careful control is needed, as *H. pluvialis* in particular will stop growing above ~30 °C, even though carotenoid biosynthesis is accelerated under such stress [32].

### 4.3. pH and Acidity/Alkalinity

Both *H. pluvialis* and *C. zofingiensis* prefer near-neutral pH conditions (i.e., medium acidity/alkalinity near neutrality), and pH can influence pigment production indirectly via its effects on cellular physiology. For *H. pluvialis*, the optimal pH for biomass growth lies in the range ~7.0–7.5 (up to about 7.8) [50]. In well-buffered cultures at pH ~7, the algae maintain high photosynthetic efficiency and chlorophyll stability.

Deviations outside this near-neutral pH range impose stress. *H. pluvialis* is known to tolerate mildly acidic or alkaline waters in nature due to its encystment capability, but laboratory studies show that high acidity (pH < 6) or high alkalinity (pH > 8) can inhibit growth and pigment synthesis [32]. Under acidic stress, chlorophyll is degraded and astaxanthin can accumulate as a protective sink for excess energy, similar to other stress conditions. For example, a brief exposure of *H. pluvialis* to extreme acidity (pH ~4.0) has been shown to trigger encystment and nearly double the cellular astaxanthin content within 12 h [51]. Indeed, *H. pluvialis* cells exposed to “high acidity” conditions exhibit metabolic symptoms of stress (e.g., starch accumulation followed by carotenoid synthesis) analogous to nutrient or light stress responses. Alkaline stress, on the other hand, may reduce nutrient availability and damage cellular structures, also leading to secondary carotenoid induction in severe cases. *C. zofingiensis* is likewise usually cultured around neutral pH; for instance, an initial pH of ~6.8–7.0 is often used in growth media [52]. Significant pH fluctuations in *C. zofingiensis* cultures (e.g., due to CO_2_ depletion or excessive bicarbonate) could act as an abiotic stress, but there has been limited focused research on pH as a trigger for astaxanthin in this species. A recent pilot-scale study noted that pH oscillations were not extensively examined as stress inducers for *C. zofingiensis* [53].

### 4.4. Salinity and Osmotic Stress

Osmotic stress induced by salt addition is a well-established trigger of astaxanthin biosynthesis. Osmotic upshift causes cellular dehydration and ionic imbalance, generating ROS and impairing photosynthesis. The alga mitigates these effects by synthesizing astaxanthin and related ketocarotenoids that quench ROS and stabilize membranes. The freshwater alga *H. pluvialis* responds strongly to elevated salinity; moderate NaCl levels (0.2–0.5% *w*/*v*, ~30–70 mM) can induce transition to the astaxanthin-accumulating stage [54]. For example, 1% NaCl (~0.17 M) for 10 days raised astaxanthin content in *H. pluvialis* from ~3.5 to 17.7 mg g^−1^ (nearly 5-fold increase) [55]. Very high salinity (>0.5 M NaCl) can further boost astaxanthin accumulation (>2-fold) [56], though extreme concentrations eventually impair growth (e.g., ≥0.6 M causes severe inhibition). *C. zofingiensis*, also a freshwater species, tolerates salinity more effectively and uses osmotic stress to promote carotenoid and lipid accumulation [57]. For instance, *C. zofingiensis* can grow at ~100 mM NaCl (~0.58% *w*/*v*) with minimal growth reduction, and astaxanthin content is significantly enhanced under ~200 mM NaCl stress [58]. In one study, a mild salt addition of 1 g L^−1^ NaCl (~17 mM) yielded an astaxanthin content of ~21.4 mg g^−1^, which increased further (to ~25–31 mg g^−1^) when salt stress was combined with nitrogen deprivation [37]. In a recent study, a two-phase regime involving nitrogen deprivation followed by 17.5 g L^−1^ NaCl (~1.75%) increased astaxanthin content from ~2.2 to 4.9 mg g^−1^ (>2-fold) [53]. Although *C. zofingiensis* accumulates less astaxanthin than *H. pluvialis*, these results confirm salinity as a strong inductive cue in both species.

### 4.5. Nutrient Availability and Starvation

Nutrient stress is one of the strongest regulators of astaxanthin biosynthesis. Both *H. pluvialis* and *C. zofingiensis* natively produce astaxanthin as a secondary carotenoid under conditions of macronutrient limitation, which curtail growth but induce a shift toward secondary metabolism. Nitrogen deprivation is paramount: *H. pluvialis* cells under nitrogen starvation stop dividing and rapidly accumulate astaxanthin-rich oil globules, turning from green to red within days [59]. The absence of nitrogen leads to loss of chlorophyll and dismantling of the photosynthetic apparatus, which exposes the cells to excess light energy and oxidative stress—direct triggers for the carotenoid pathway. Studies have quantified that nitrogen limitation induces astaxanthin at roughly double the rate of phosphorus limitation in *H. pluvialis*, reflecting the more severe cellular damage and ROS generation when protein synthesis is halted by nitrogen lack [60]. Phosphate limitation also prompts astaxanthin accumulation, but typically to a lesser extent, likely because P-starved cells can still maintain some photosynthetic function and suffer less photooxidative damage than nitrogen-starved cells. Sulfur deprivation and other nutrient stresses (e.g., magnesium deficiency) have similarly been reported to induce astaxanthin in *H. pluvialis*, though these are less commonly applied than nitrogen or phosphorus stress. In *C. zofingiensis*, nutrient stress elicits a comparable response: nitrogen or phosphorus starvation causes a decrease in cell division and chlorophyll, and initiates astaxanthin accumulation together with other storage compounds. For instance, under nitrogen starvation *C. zofingiensis* enters a “chromatic acclimation” where chlorophyll levels drop and carotenoids (including astaxanthin and β-carotene) increase, giving cells a yellow-orange hue [52]. Transcriptomic analyses in *C. zofingiensis* confirm upregulation of carotenogenesis genes during nitrogen starvation, many of which overlap with the high-light stress response [33]. Interestingly, *C. zofingiensis* tends to accumulate relatively more astaxanthin per cell under nitrogen-starved phototrophic conditions than under heterotrophic conditions. In fully heterotrophic (dark, sugar-fed) culture with nitrogen starvation, *C. zofingiensis* astaxanthin content may reach only ~0.06% DW, whereas nitrogen-starved cells exposed to light accumulate around 0.5% DW [61]. Light provides additional energy and ROS stimulus to drive the astaxanthin pathway. As a result, an effective strategy for *C. zofingiensis* is two-stage cultivation: first, grow the cells with sufficient nitrogen (and possibly in the dark or low light to concentrate biomass), then impose nitrogen deprivation and illuminate to induce astaxanthin. This mirrors the well-established two-stage process in *H. pluvialis*, where a nutrient-replete “green stage” is followed by a nitrogen-starved “red stage”. During such stress phases, profound metabolic remodeling occurs in both algae. Carbon flux is redirected from protein synthesis into storage carbohydrates and lipids, which serve as carbon sinks and scaffolds for astaxanthin esterification [52]. In *H. pluvialis*, up to 40–70% of the cell’s DW can become starch and oil during prolonged nitrogen starvation [32], after which astaxanthin is esterified and deposited in oil droplets. *C. zofingiensis* shows the same trend: in one study, nitrogen-starved cells accumulated ~50% DW as starch by day 19 of stress, then progressively converted some of that carbohydrate into lipids (reaching ~25% lipid DW) as carotenoid synthesis ramped up [53]. This indicates a coordinated stress response where initial energy (as starch) is later channeled into lipid-linked secondary products like astaxanthin. Importantly, nutrient stress often works best in combination with other factors (light, salt) to maximize astaxanthin. For example, *H. pluvialis* under nitrogen starvation and high light attains much higher astaxanthin levels than under nitrogen starvation alone [6]. Similarly, *C. zofingiensis* can show only modest astaxanthin under pure nitrogen starvation in dark conditions, but nitrogen starvation plus illumination or osmotic shock yields a significantly stronger carotenoid response [57].

## 5. Engineering and Bioprocess Strategies

### 5.1. Cultivation Systems

A variety of large-scale microalgae cultivation systems are available to accommodate different requirements for light and nutrient supply. Among them, the open raceway pond (ORP) utilizes natural sunlight for algal cultivation. For instance, *H. pluvialis* has been successfully cultured in ORP systems to induce astaxanthin accumulation, achieving a yield of 140 mg m^−2^ d^−1^ [62]. In addition, *C. zofingiensis* is typically cultivated in ORPs for purifying dairy wastewater with low cell densities at 9.05 × 10^6^ cells mL^−1^ [63]. The ORPs suffer from several drawbacks, including uncontrollable light intensity, rapid water loss, and susceptibility to seasonal, weather, and microbial contamination, making them unsuitable for *C. zofingiensis* cultivation.

Closed photobioreactors (PBRs) include tubular, flat-panel, and column types, all made of transparent materials with high surface-to-volume ratios. These systems commonly employ light-emitting diodes (LEDs) as light sources and are equipped with advanced aeration, temperature control, and sterilization systems. Such setups overcome most limitations of open RPs and provide a stable, controllable environment for microalgae cultivation, meeting the demands of various algal species, nutritional modes, and production cost constraints. Compared to open ponds, the average biomass concentration in tubular reactors can increase to 5 g L^−1^ [64]. In tower-type PBRs, *H. pluvialis* cultures can accumulate astaxanthin up to 25.92 mg L^−1^ [65]. Wood et al. (2023) cultivated *C. zofingiensis* in a 65 L PBR under nitrogen-limited and high-light induction conditions, obtaining an astaxanthin yield of 14.7 mg L^−1^ on day 8 [66].

The fermenter is another commonly used cultivation system in industrial microalgal production [67]. Its main advantage lies in the ability to stably supply organic carbon or other nutrients according to preset parameters, making it suitable for non-batch cultivation strategies focused on cell density accumulation. Fermenters can be adapted for phototrophic culture by incorporating external light sources; however, light distribution is generally less uniform than in PBR systems, leading to substantial heterogeneity in photon absorption and significant light energy waste. Despite this, fermenters allow precise control over cultivation conditions and nutrient inputs, facilitating heterotrophic biomass accumulation. In most cases, they are preferred for heterotrophic culture. Furthermore, fermenters offer advantages such as finely tunable internal conditions (temperature, aeration, liquid volume, and pH), tolerance to steam sterilization, large capacity with simple configuration, ease of product harvesting and in situ cleaning, and low operating and energy costs—features that make them well-suited for industrial-scale fermentation processes.

### 5.2. Cultivation Strategy

The two-stage phototrophic cultivation process of *H. pluvialis* remains the most validated approach for achieving ultra-high astaxanthin yields (Table 2). During the green stage, cells are maintained under optimal growth conditions (20–25 °C, pH 7.0–7.5, 300–500 µmol photons m^−2^ s^−1^) with nitrate concentrations of 8–17 mM, enabling biomass densities of 4–6 g DW L^−1^ within 8–10 days. Transition to the red stage is induced through combined stresses: nitrate depletion (≤1 mM), elevated salinity (1.5–2% NaCl), and increased light intensity (800–1500 µmol photons m^−2^ s^−1^) [26,68]. For instance, optimization of fed-batch cultivation and transformation conditions in a 5-L photobioreactor resulted in a maximum dry cell weight of 1.87 g L^−1^—approximately double that of batch cultivation. Exposure to mixed blue and white light (3:1 ratio) at 7000 lx further accelerated encystment, yielding 91.8 mg L^−1^ of astaxanthin within a shortened cultivation period [6]. Moreover, elevated carbon-to-nitrogen (C/N) ratios markedly enhanced acetyl-CoA generation and redirected carbon flux toward astaxanthin biosynthesis. A C/N-gradient fed-batch strategy subsequently achieved 9.18 g L^−1^ of biomass consisting entirely of immotile cyst cells, with an astaxanthin productivity of 15.45 mg L^−1^ d^−1^—demonstrating strong potential for industrial application [69]. Continuous one-stage chemostat versions of this approach have also been implemented in 20 mm flat-panel photobioreactors operated at a dilution rate of 0.015 h^−1^. By maintaining a mean rate of photon absorption (MRPA) of 9000 µmol m^−2^ s^−1^, astaxanthin productivity reached 1.27 ± 0.03 g m^−2^ d^−1^ (≈190 mg L^−1^ d^−1^), with intracellular content of 3.9% DW [68]. Despite these advances, large-scale implementation remains constrained by photo-inhibition, thermal stress, and contamination risks.

*C. zofingiensis* offers a rapid and efficient alternative route for astaxanthin production owing to its capacity for high-density heterotrophic growth. During the heterotrophic phase, cells typically reach biomass concentrations of 100–235 g L^−1^ (DW), with lipid contents exceeding 40%, while astaxanthin synthesis remains repressed [3]. Following this phase, the culture is diluted to 10–50 g L^−1^ and exposed to strong illumination to initiate astaxanthin biosynthesis [8]. When combined blue-light and chemical induction (gibberellin A_3_, 2 mg L^−1^; H_2_O_2_, 5 mL L^−1^) were applied after heterotrophic biomass accumulation, carbon flux was redirected toward carotenoid metabolism, enabling *C. zofingiensis* to accumulate up to 6.26 mg g^−1^ astaxanthin with a productivity of 39 mg L^−1^ d^−1^ [8]. Similarly, an optimized two-stage process incorporating gibberellic acid (GA_3_), 0.5 g L^−1^ Fe^2+^, and 0.1% corn steep liquor achieved 318 mg L^−1^ total astaxanthin within 24–48 h, corresponding to productivities of 110–130 mg L^−1^ d^−1^ and a cellular astaxanthin content of 0.144% DW [3]. This represents a 4–5-fold increase over the optimal yield of *H. pluvialis*, with biomass conversion efficiency reaching ~1.3% (*w*/*w*) [70]. In contrast, the heterotrophy–photoinduction strategy of *C. zofingiensis* enables rapid astaxanthin accumulation without extensive light dependence during biomass build-up, providing a major advantage for large-scale production under conditions with readily available heterotrophic substrates [71].

**Table 2:** Representative engineering strategies enhancing astaxanthin production in microalgae.

Strategy Type	Specific Approach	Improvement	Reference
Cultivation optimization	Two-stage light + N-limitation (*H. pluvialis*)	15.45 mg L^−1^ d^−1^ productivity; 3.9% DW astaxanthin	[68,69]
Light regime	Mixed blue:white (3:1) at 7000 lx	91.8 mg L^−1^ astaxanthin yield	[6]
Two-stage heterotrophy–photoinduction	Glucose-fed fermentation → high-light induction (*C. zofingiensis*)	6.26 mg g^−1^ cellular astaxanthin; 39 mg L^−1^ d^−1^	[8]
Chemical stimulation	GA_3_ (2 mg L^−1^) + Fe^2+^ (0.5 g L^−1^) + corn steep liquor (0.1%)	318 mg L^−1^ astaxanthin (4–5× increase)	[3]
Genetic engineering	Overexpression of PSY/BKT and down-regulation of LCYe	Enhanced carotenoid flux → higher astaxanthin biosynthesis	[31,72]
Lipid-droplet engineering	Co-expression of MLDP and DGAT1	Improved esterification and storage capacity	[73,74]
Green extraction	Supercritical CO_2_ (+20% ethanol co-solvent)	>90% recovery, solvent-free product	[75,76]

### 5.3. Metabolic and Genetic Engineering

Precise genetic editing technologies, such as CRISPR–Cas9, have opened new avenues to enhance astaxanthin biosynthesis. Common engineering strategies focus on boosting rate-limiting enzymes and blocking competing pathways (Table 2). For instance, genetically overexpressing key chloroplastic carotenoid biosynthetic enzymes—such as PSY, which initiates the pathway, and BKT, a key enzyme for astaxanthin formation—can increase carbon flux toward astaxanthin precursors. Such overexpression is typically achieved via metabolic engineering (e.g., using strong promoters or multiple gene copies), and has yielded 2–3-fold increases in carotenoid production when *PSY* or *BKT* levels are elevated [77]. Overexpression of PDS has been shown to enhance carotenoid biosynthesis in several algal species, including *C. zofingiensis* [78], *H. pluvialis* [79], and *C. reinhardtii* [80]. Meanwhile, suppressing enzymes that divert flux into alternative branches, such as LCYe, which catalyzes α-carotene and lutein formation, effectively minimizes metabolic competition [31,72]. This down-regulation of *LCYe* can be implemented through targeted gene knockouts or RNA interference; for example, a CRISPR/Cas9 knockout of *LCYE* in *C. reinhardtii* redirected flux from lutein to β-carotene, resulting in ~2.3-fold higher astaxanthin accumulation [81]. Moreover, under astaxanthin-inducing stress conditions (e.g., high light), *PSY* and *BKT* transcripts are naturally upregulated while *LCYe* expression is repressed, further channeling carbon toward ketocarotenoids [82]. In parallel, strengthening the MEP pathway through overexpression of its key enzymes—DXS, DXR, and HDR—enhances the supply of the isoprenoid precursors IPP and DMAPP [31]. Similarly, amplifying enzymes from GGPP through lycopene diverts IPP away from sterol or terpenoid synthesis toward carotenoids [83]. Together, these genetic interventions synergistically increase metabolic flux, improve precursor availability, and reduce accumulation of intermediates.

Lipid droplet engineering represents another frontier for enhancing astaxanthin yield [74]. Co-expression of lipid droplet–associated proteins (such as the major lipid droplet protein, MLDP, or its homologs) with astaxanthin esterification enzymes enhances the anchoring and storage capacity of lipid droplets for hydrophobic astaxanthin esters [73]. This not only mitigates feedback inhibition and oxidative degradation within the cell but also expands the cellular capacity for astaxanthin accumulation. Higher esterification efficiency enables astaxanthin to be stably stored in esterified form, thereby improving product stability and downstream recovery efficiency.

Organelle compartmentalization offers an additional strategy to optimize pathway efficiency. In algal cells, primary carotenoid synthesis (up to β-carotene) occurs in the chloroplast, whereas the later ketolation and hydroxylation steps to form astaxanthin happen in the cytosol or at the ER–lipid droplet interface. For example, one can target early steps (like IPP generation and carotene synthesis) to the chloroplast—tapping into its high levels of precursors and efficient isoprenoid enzymes—while re-routing the final steps (BKT and β-carotene hydroxylase CHYb reactions) to the cytosol or ER, near the sites of lipid-droplet formation [34]. Such organelle-specific expression can reduce metabolic crosstalk and competition for resources. By isolating the astaxanthin pathway from other plastidial processes (and from sensitive feedback controls in the chloroplast), the cell can convert intermediates to astaxanthin more efficiently [84]. By mimicking and enhancing these spatial separations in microalgae, researchers aim to maximize astaxanthin output while minimizing interference from other metabolic pathways. Notably, *H. pluvialis* appears to naturally export β-carotene out of the chloroplast for astaxanthin formation in the cytosol, as both BKT and CHYb enzyme activities have been detected in isolated lipid droplets of this alga [34,73]. Facilitating the transport of carotenoid intermediates (β-carotene, zeaxanthin) from the chloroplast to the external compartments is therefore another key consideration. Recent studies even suggest a budding mechanism from the chloroplast to shuttle β-carotene into the cytoplasm [34,85]. The spatial segregation reduces metabolic competition and enhances substrate utilization, thereby increasing overall productivity [86].

### 5.4. Extraction and Downstream Processing

Since microalgae have a robust and resistant cell wall, effective cell disruption is essential to release and recover astaxanthin efficiently. The choice of disruption and extraction method greatly affects yield, purity, and pigment stability.

Bead milling is a mechanical grinding method where micro-sized beads physically rupture algal cells through collisions. Incorporating a bead-milling step before extraction has been shown to boost astaxanthin recovery to ~92% [87]. Mild bead milling (using food-grade solvents like ethanol or acetone) can greatly improve solvent access to astaxanthin, though excessive milling can generate heat and cause some pigment degradation [88]. High-pressure homogenization (HPH) forces a concentrated *H. pluvialis* slurry through a narrow valve at pressures of 70–80 MPa, shearing the cells. HPH can achieve over 90% cell disruption after 1–2 passes [89], enabling high astaxanthin yields. HPH generates some heat (e.g., ~2 °C per 10 MPa increase in these trials), but short exposure under controlled conditions did not significantly degrade or isomerize astaxanthin. Ultrasound-assisted extraction (UAE) is commonly employed for preliminary cell disruption: cavitation generates instantaneous high temperatures, high pressures and micro-jets, which create numerous pores and enhance solute diffusion, thereby efficiently disrupting cell walls at ambient temperature [90]. Studies have shown that for *H. pluvialis* pre-treated by UAE, the naked astaxanthin release rate can exceed 90% [91]. This low-temperature, high-efficiency cell-breakage method avoids thermal degradation of astaxanthin and accelerates downstream extraction. Enzymatic cell-wall lysis uses hydrolytic enzymes (e.g., cellulase, pectinase) to digest the robust polysaccharide wall of *H. pluvialis*, thereby freeing astaxanthin at mild conditions. Enzyme pretreatment can significantly improve astaxanthin release. For instance, pectinase yielded about 75.3% astaxanthin recovery, outperforming cellulase (~67.1% yield) under optimal conditions [92]. Enzymatic methods preserve astaxanthin’s stability and avoid harsh chemicals, but they are slower and typically less efficient or scalable than mechanical disruption [93]. SFE-CO_2_ is the preferred industrial method for purification of natural astaxanthin. By adjusting temperature, pressure and using a low-toxicity co-solvent (e.g., ethanol), SFE-CO_2_ enables high-efficiency recovery with no solvent residues. For example, under moderate conditions (~40–55 °C, 300–550 bar) with ~20% ethanol co-solvent, recoveries of >90% astaxanthin have been achieved [75]. Compared to conventional organic-solvent extraction, SFE-CO_2_ is non-toxic, solvent-free and fulfils food-grade “no residual solvent” requirements. Innovative micro-fluidic SFE chip systems further reduce pressure requirements (≈8–15 MPa); for instance, a microfluidic reactor at 55 °C and 8 MPa achieved ~92% astaxanthin recovery, and with ethanol co-solvent the extraction time fell from 15 h to mere seconds [76].

A comparative evaluation of disruption strategies therefore highlights species-specific optimality: (i) HPH and bead milling are favored for *H. pluvialis* due to the high mechanical resistance of its cyst wall; (ii) UAE and enzymatic disruption are better suited for *C. zofingiensis,* balancing lower energy requirements with high pigment recovery. Furthermore, SFE-CO_2_ remains widely applicable for both algae as a downstream purification step, but its overall efficiency is contingent on sufficient pre-disruption of *H. pluvialis* biomass. Incorporating cell wall characteristics into method selection can significantly reduce energy consumption, improve astaxanthin recovery, and enhance techno-economic feasibility for each species.

Because astaxanthin is highly hydrophobic, oil-in-water (O/W) nano-emulsions and micro-encapsulation technologies have been widely studied to improve its aqueous dispersibility and bioavailability. For example, whey-protein-based O/W nano-emulsions with algal oil as the oil phase have demonstrated significantly enhanced cellular uptake and stability of astaxanthin [94]. Nanostructured lipid carriers (NLCs) have achieved recoveries greater than 90% and improved intestinal permeability [95]. Review studies further underline that encapsulation (nano/micro) markedly improves astaxanthin’s stability, release profile, and bio-accessibility in vitro and in some cases in vivo [96]. These formulation strategies—whether high-pressure homogenized nano-emulsions, micro-capsules (e.g., starch/gelatin) or NLCs—therefore provide a promising route to enhance astaxanthin stability, sustained release, oral bioavailability and the viability of applications in food, cosmetic and functional feed sectors.

## 6. Industrial Potential and Techno-Economic Analysis

*H. pluvialis* and *C. zofingiensis* exhibit sharply contrasting profiles in large-scale astaxanthin production (Table 3). *H. pluvialis* achieves an exceptionally high intracellular astaxanthin content but grows slowly, typically reaching only ~5–7 g L^−1^ dry cell weight even after extended cultivation. In contrast, *C. zofingiensis* can rapidly attain ultrahigh cell densities approaching 50–220 g L^−1^ under optimized fed-batch or heterotrophic conditions [11,37]. This order-of-magnitude difference in biomass yield means that, despite *H. pluvialis* having richer cellular astaxanthin, the volumetric astaxanthin productivity of *C. zofingiensis* can equal or exceed that of *H. pluvialis*. Such performance allows *C. zofingiensis* to reach comparable astaxanthin yields in a fraction of the cultivation time, translating to more frequent harvesting and higher annual output. Additionally, *C. zofingiensis* is intrinsically more robust across culture modes: it grows under both photoautotrophic and heterotrophic regimes, whereas *H. pluvialis* relies on a slower two-stage photoinduction process. This flexibility streamlines production by enabling a fast biomass build-up in the dark followed by a brief light-induced astaxanthin accumulation, thereby further shortening the production cycle without compromising pigment titers.

From a techno-economic perspective, the high productivity and density of *C. zofingiensis* confer significant cost advantages. Dense cultures greatly reduce downstream costs: harvesting and dewatering represent a major expense in microalgal farms, often ~20–30% of total cost, but *C. zofingiensis* can cut this fraction by yielding far more biomass (and astaxanthin) per unit volume [97]. By reaching ~10–15 times the cell density of *H. pluvialis*, *C. zofingiensis* minimizes the volume of culture to process for the same astaxanthin output, saving energy and infrastructure in centrifugation, drying, and extraction. Moreover, *C. zofingiensis* can be cultivated heterotrophically in standard fermentors, which typically have lower capital and operating costs per liter than the large-area photobioreactors or open ponds required for *H. pluvialis*. Phototrophic *H. pluvialis* production demands extensive light exposure systems (tubular PBRs or raceway ponds) and careful environmental control, driving up capital expenditure and operating costs. In contrast, heterotrophic cultivation of *C. zofingiensis* foregoes expensive lighting and land footprint, instead leveraging cheaper sugar substrates and existing fermentation technology. The trade-off is the cost of organic carbon feed, but this can be mitigated by using low-cost feedstocks (e.g., industrial glucose or waste glycerol), and is offset by much faster biomass accumulation. Techno-economic analyses indicate that current natural astaxanthin sells for $2500–7000/kg, reflecting *H. pluvialis*’ laborious production [21]. However, optimized processes have demonstrated the potential to lower production costs dramatically: one pilot-scale assessment of *H. pluvialis* estimated astaxanthin costs could be reduced to ~$718/kg at scale [98], approaching the cost of synthetic astaxanthin. With *C. zofingiensis*, similar or greater cost reductions are expected due to its superior biomass productivity and co-product synergy. By delivering more astaxanthin per batch (and per reactor), capital payback is faster and unit production costs drop. Additionally, *H. pluvialis* cultivation suffers from frequent contamination risks—notably devastation by the parasitic fungus *Paraphysoderma*—which can wipe out cultures and inflate costs. *C. zofingiensis* is also susceptible to this pathogen but is less frequently afflicted, and its use diversifies the industrial portfolio, building resilience against such crop failures [99].

Environmental and sustainability metrics likewise favor *C. zofingiensis* in several respects. Life-cycle assessments (LCA) highlight that microalgal astaxanthin production can be resource-intensive, particularly in water and energy use, but *C. zofingiensis* offers opportunities to improve these factors. *H. pluvialis* farms typically require large volumes of clean water and nutrients, and in warm climates substantial water is lost to evaporation in open ponds. By contrast, *C. zofingiensis* can thrive in closed fermentative systems or utilize alternative water sources, markedly reducing the freshwater footprint [100]. Notably, recent studies explore cultivating both species on wastewater or industrial effluents to recycle nutrients and water. This wastewater-integrated approach has dual benefits: it supplies *C. zofingiensis* with nitrogen and phosphorus for growth while simultaneously remediating the wastewater, thereby promoting a circular economy [100,101]. Using secondary-treated water or effluents in place of freshwater can curtail the overall water demand and pollution load of production. *C. zofingiensis*’ versatility (growing photoautotrophically on CO_2_ or mixotrophically on organic carbon) also means it can be coupled with waste CO_2_ streams or organic waste valorization, potentially lowering the carbon footprint of astaxanthin manufacture. High-density cultivation further translates to less land use per kg astaxanthin produced, which mitigates habitat and resource impacts. In terms of energy, *C. zofingiensis*’ softer cell wall (especially in non-cyst, heterotrophic form) can simplify cell disruption and extraction compared to the thick-walled *H. pluvialis* cysts, reducing energy input in downstream processing. Overall, each kilogram of astaxanthin from *C. zofingiensis* is poised to embody a smaller environmental burden when advanced culture techniques are applied. Both species stand to gain from sustainable innovations (e.g., solar-powered systems, nutrient recycling), but the inherently shorter cultivation cycle and multi-feedstock capability of *C. zofingiensis* give it an edge in minimizing water use, waste generation, and greenhouse gas emissions per unit of product [100,102].

Another key advantage of *C. zofingiensis* lies in product flexibility and downstream valorization. This alga has been widely cited as an ideal candidate for microalgal biorefineries because it can accumulate not only astaxanthin but also significant co-products such as lipids, proteins, and carbohydrates under suitable conditions [99]. The concurrent synthesis of astaxanthin and energy-dense lipids means a *C. zofingiensis* culture can generate multiple value streams from the same biomass. In a single batch, astaxanthin can be extracted for high-value nutraceutical or feed applications, while the residual oil-rich biomass can be converted to biodiesel or supplements, and leftover proteins and polysaccharides can serve as animal feed or fertilizer [99]. Regarding regulatory and commercial readiness, *H. pluvialis* currently dominates the natural astaxanthin market and its biomass/extracts have GRAS status or equivalent approvals in many regions, having a decades-long safety record. *C. zofingiensis, however,* still faces significant regulatory hurdles before gaining approval for feed or food applications, pending comprehensive safety and production assessments.

## 7. Technological Advancements and Patents in Astaxanthin Production

The industrial-scale astaxanthin production from *H. pluvialis* and *C. zofingiensis* has been accelerated by cutting-edge innovations across genetics, cultivation, and downstream processing. For genetic engineering, CRISPR/Cas9 genome editing has been successfully employed to enhance astaxanthin biosynthesis. For example, knocking out *bkt1* and *crtZ* in *C. zofingiensis* yielded mutants with significantly higher astaxanthin accumulation [103]. Although *H. pluvialis* has historically been recalcitrant to genetic modification, recent advances in transformation (e.g., biolistic integration of endogenous gene cassettes) now enable stable expression of foreign genes [104], opening the door to metabolic engineering in this alga. These efforts are bolstered by new genomic resources—a 2023 chromosome-level assembly of *H. pluvialis* identified [105]. Likewise, key pathway genes in *C. zofingiensis* have been elucidated and patented, providing a blueprint for strain improvement [106]. Regulatory and public acceptance barriers can significantly limit the near-term industrial deployment of genetically engineered microalgae, particularly for food and nutraceutical applications. While CRISPR-edited strains are technically promising, regulatory uncertainty and consumer acceptance mean that their initial adoption is more likely in closed systems or non-food uses, with broader deployment depending on future regulatory harmonization and public perception.

Given this increasing integration of biological innovation and intellectual property, a comprehensive overview of recently issued patents—covering chemical synthesis, microbial and algal fermentation, metabolic engineering, and high-density cultivation—is summarized in Table 4 to illustrate current technological trends and global patent activity. The novel bioreactor designs are also emerging: a patented floating photobioreactor (RFP) that relies on passive rotation was used in outdoor *C. zofingiensis* culture to efficiently induce astaxanthin accumulation with minimal energy input [10]. On the industrial front, cost-oriented process innovations are being protected by patents—for instance, a recent patent from Russia outlines replacing expensive nitrate media with urea in *C. zofingiensis* cultivation to cut nutrient costs while co-producing lipids and astaxanthin (RU2715039C1). Finally, downstream processing has likewise advanced toward sustainability and efficiency. SFE-CO_2_, as described in Chinese patent CN101691348A, enables solvent-free recovery of astaxanthin from dried *H. pluvialis* biomass with minimal loss of activity, although high equipment costs persist. In China, companies and institutes overwhelmingly patent process/scale innovations (bioreactor scale-up, medium formulations, illumination regimes, fed-batch feeding). In the U.S., patents often blend these with biotech—protecting engineered strains or algae extracts (e.g., genes from *C.* for astaxanthin overproduction). European patents (and PCTs with EU applicants) frequently target cultivation efficiency (e.g., novel photobioreactor configurations) and sustainable processing, reflecting a priority on “green” production. From a freedom-to-operate (FTO) perspective, the current astaxanthin patent landscape is relatively crowded but not fully closed. Core patents related to *H. pluvialis* cultivation, extraction, and formulation are largely held by established players, which can constrain direct imitation of mature industrial processes. However, opportunities for FTO remain through alternative process integration, cultivation strategies, strain selection, and downstream optimization, particularly for emerging platforms such as *C. zofingiensis*, where patent coverage is still evolving and less consolidated.

## 8. Conclusions

Astaxanthin-producing microalgae, particularly *H. pluvialis* and *C. zofingiensis*, continue to show strong potential as sustainable sources of high-value bioproducts. Advances in light management, nutrient modulation, and photobioreactor design have improved biomass and pigment yields, while molecular tools have enabled more targeted regulation of carotenoid biosynthesis. These developments support the integration of astaxanthin production into broader biorefinery platforms. Despite progress, challenges such as high production costs, strain sensitivity, and processing inefficiencies remain. Regulatory approval, especially for *C. zofingiensis*, also requires further safety validation. This study highlights the scientific relevance of combining cultivation and genetic strategies to enhance biosynthetic output. Astaxanthin’s broad applications in nutraceuticals, cosmetics, and pharmaceuticals—owing to its antioxidant and anti-inflammatory properties—further underline its commercial appeal. Future work should focus on improving strain robustness, scaling continuous processes, and assessing sustainability and economic feasibility to support industrial translation.

## Figures and Tables

**Table 1:** Astaxanthin Biosynthesis Regulation in *H. pluvialis* vs. *C. zofingiensis*.

Aspect	*H. pluvialis*	*C. zofingiensis*
MEP pathway regulation	MEP genes are upregulated under stress, enhancing precursor supply	MEP genes show no stress-induced upregulation
Astaxanthin biosynthesis route	Uses both canthaxanthin hydroxylation and zeaxanthin ketolation	Relies only on zeaxanthin ketolation; cannot convert canthaxanthin
BKT/CHYb enzyme activity	High efficiency with minimal intermediate buildup	Limited activity; accumulates intermediates
Carbon flux and yield	Strong carotenoid flux; astaxanthin up to 3–5% DW	Weaker flux; astaxanthin typically 0.1–0.5% DW
Competing branches	No keto-lutein; violaxanthin synthesis suppressed under stress	Produces keto-lutein; zeaxanthin diverted to violaxanthin
Esterification and storage	Astaxanthin esterified in lipid droplets; depends on TAG synthesis	Storage less TAG-dependent; esterification shared with other pigments

**Table 3:** Comparative physiological and bioprocess characteristics of *H. pluvialis* and *C. zofingiensis*.

Parameter	*H. pluvialis*	*C. zofingiensis*
Growth mode	Obligately photoautotrophic	Photoautotrophic, mixotrophic, or heterotrophic
Maximum biomass concentration	5–10 g L^−1^	100–220 g L^−1^ (heterotrophic fed-batch)
Maximum astaxanthin content (cellular)	3–5% DW	0.1–0.5% DW
Cultivation cycle	10–15 days	5–8 days
Optimal temperature	20–25 °C (growth arrest > 30 °C)	25–27 °C (tolerates mild heat)
Optimal pH	7.0–7.5	6.8–7.0
Inductive stresses	High light, N/P starvation, salinity	Nitrogen starvation + illumination or osmotic stress
Cell wall and extraction	Thick-walled cysts; high mechanical energy needed	Thin-walled cells; easier extraction
Typical cultivation strategy	Two-stage phototrophic induction (“green–red” process)	Heterotrophic biomass build-up → photoinduction
Industrial potential	Highest pigment purity but costly and light-dependent	Low-cost, flexible, co-production of lipids and astaxanthin

**Table 4:** Representative patents related to astaxanthin synthesis, microbial fermentation, and algal engineering.

Patent No.	Title	Technical Advantages	Production Mode	Organism/Substrate	Yield/Titer	Key Conditions	Legal Status	Country/Region
WO 2024/200695 A1	Improved process for the production of an astaxanthin intermediate	Non-halogenated solvents; avoids alkyl-lithium reagents by using Grignard reagents; higher stability	Chemical synthesis	Non-halogenated solvents (ethers, alkanes)	Up to 91%	Three-step synthesis	PCT application	DSM IP Assets B.V., Netherlands
WO 2024/261003 A1	New astaxanthin synthesis	Halogen-free solvents; separable intermediates; environmentally safer process	Chemical synthesis	Hydrocarbons/carbonates	Overall yield up to 85%	Non-halogenated solvent system	PCT application	DSM IP Assets B.V., Netherlands
WO 2025/099193 A1	Improved process for the production of an astaxanthin intermediate	Low-temperature operation (≥0 °C); energy saving	Chemical synthesis	Ether solvents (e.g., THF)	82.8%	Three-step synthesis	PCT application	DSM IP Assets B.V., Netherlands
CN 116217454 B	Method for preparing astaxanthin	Valorization of vitamin A acetate crystallization waste; resource-efficient pathway	Chemical synthesis	Vitamin A acetate waste liquor	Overall yield 80%	Four-step synthesis route	Granted	China (Wanhua Chemical Group)
CN 115385837 B	One-step oxidation to produce astaxanthin from canthaxanthin	Mild reaction; simplified process	Chemical synthesis	Canthaxanthin	80%	Low-temperature oxidation	Granted	China (Wanhua Chemical Group)
WO 2024/001460 A1	Method and vector for biosynthesis of astaxanthin	G135L point mutation and protein–scaffold system boosting pathway flux	Microbial fermentation	*Yarrowia lipolytica*	~3× vs. control	Fermenter cultivation	PCT application	Wuhan Hesheng Technology Co., Ltd. (China)
WO 2025/103054 A1	High-carotenoid *Yarrowia lipolytica* and applications	Xylose-inducible expression system enabling controlled biosynthesis	Microbial fermentation	Engineered *Y. lipolytica*	112.86 mg L^−1^	High-density fermentation; xylose induction	PCT application	East China University of Science & Technology
CN 120624591 A	Fermentation-based astaxanthin production by *Phaffia rhodozyma*	Two-step seed activation improves cell viability	Microbial fermentation	*Phaffia rhodozyma*	92.9–94.5 mg L^−1^	Fed-batch at 20 °C	Published	China (Hainachuan Biotech)
CN 108913746 A	Enhanced astaxanthin synthesis in *P. rhodozyma*	Tomato-powder supplementation increases biomass and pigment	Microbial fermentation	*Phaffia rhodozyma*	81.76 mg L^−1^	Fed-batch; pH control	Granted	China (Weihai Lida Biotech)
WO 2020/103622 A1	Induction strategy for concurrent astaxanthin and lipid accumulation in *C. zofingiensis*	Phytohormone-induced co-accumulation of oil and astaxanthin	Algal fermentation	*C. zofingiensis*	Biomass 98 g L^−1^; astaxanthin 13.1 mg g^−1^; lipids 64.5% DW	High light + N deprivation	PCT application	South China University of Technology
CN 118374567 B	Natural astaxanthin production using *C. zofingiensis*	Two-stage pH regulation enabling high-density heterotrophic fermentation	Heterotrophic fermentation	*C. zofingiensis*	500–580 mg L^−1^	Fed-batch; pH-shift induction	Published	China (Institute of Hydrobiology, CAS)
CN 108624507 B	*Chlorella* W7 capable of astaxanthin production	Mixotrophic growth; high-light induction	Algal fermentation	*Chlorella sp.* W7	1.1–2.3% DW	Nitrogen deprivation + high light	Granted	China (Wuhan AlgaeBio)
CN 114891637 B	High-producing *Chlorella zofingiensis* mutant	Stable mutant strain (12C10) with elevated astaxanthin levels	Algal fermentation	Mutant *C. zofingiensis*	+74% vs. WT	Cultured in Endo medium	Granted	China (Demeter Biotech, Zhuhai)
WO 2024/026963 A1	Construction of astaxanthin pathway in *Chlamydomonas reinhardtii*	Chloroplast engineering with multi-gene overexpression	Genetic engineering	*C. reinhardtii*	37.63 µg mg^−1^ DW (65% improvement vs. WT)	Photoautotrophic culture	PCT application	Shenzhen University

## Data Availability

The data underlying the findings of this study can be obtained from the corresponding author upon reasonable request.
